# Translating atherosclerosis research from bench to bedside: navigating the barriers for effective preclinical drug discovery

**DOI:** 10.1042/CS20210862

**Published:** 2022-12-02

**Authors:** Lauren T. May, Belinda A. Bartolo, David G. Harrison, Tomasz Guzik, Grant R. Drummond, Gemma A. Figtree, Rebecca H. Ritchie, Kerry-Anne Rye, Judy B. de Haan

**Affiliations:** 1 Drug Discovery Biology, Monash Institute of Pharmaceutical Sciences, Monash University, Parkville, Victoria 3052, Australia; 2 Faculty of Medicine and Health, University of Sydney, Sydney, Australia; 3 Division of Clinical Pharmacology, Department of Medicine, Vanderbilt University Medical Center, Nashville TN, U.S.A.; 4 Institute of Cardiovascular and Medical Sciences, University of Glasgow, U.K.; 5 Department of Medicine, Jagiellonian University Medical College, Krakow, Poland; 6 Centre for Cardiovascular Biology and Disease Research, Department of Microbiology, Anatomy, Physiology and Pharmacology, La Trobe University, Melbourne, Victoria, Australia; 7 Kolling Research Institute, University of Sydney, Sydney, Australia; 8 Imaging and Phenotyping Laboratory, Charles Perkins Centre and Faculty of Medicine and Health, University of Sydney, Sydney, Australia; 9 Drug Discovery Biology, Monash Institute of Pharmaceutical Sciences, Monash University, Parkville, Victoria, Australia; 10 Lipid Research Group, School of Medical Sciences, Faculty of Medicine, University of New South Wales, Sydney 2052, Australia; 11 Cardiovascular Inflammation and Redox Biology Lab, Baker Heart and Diabetes Institute, Melbourne, Victoria, Australia; 12 Department of Chemistry and Biotechnology, Swinburne University of Technology, Hawthorn, Victoria 3122, Australia; 13 Department Cardiometabolic Health, University of Melbourne, Parkville, Victoria 3010, Australia; 14 Department of Physiology, Anatomy and Microbiology, La Trobe University, Bundoora, Victoria 3086, Australia; 15 Department of Immunology and Pathology, Central Clinical School, Monash University, Melbourne, Victoria 3004, Australia

**Keywords:** atherosclerosis, cardiovascular disease, coronary artery disease, drug discovery and design, model organisms

## Abstract

Cardiovascular disease (CVD) remains the leading cause of death worldwide. An ongoing challenge remains the development of novel pharmacotherapies to treat CVD, particularly atherosclerosis. Effective mechanism-informed development and translation of new drugs requires a deep understanding of the known and currently unknown biological mechanisms underpinning atherosclerosis, accompanied by optimization of traditional drug discovery approaches. Current animal models do not precisely recapitulate the pathobiology underpinning human CVD. Accordingly, a fundamental limitation in early-stage drug discovery has been the lack of consensus regarding an appropriate experimental *in vivo* model that can mimic human atherosclerosis. However, when coupled with a clear understanding of the specific advantages and limitations of the model employed, preclinical animal models remain a crucial component for evaluating pharmacological interventions. Within this perspective, we will provide an overview of the mechanisms and modalities of atherosclerotic drugs, including those in the preclinical and early clinical development stage. Additionally, we highlight recent preclinical models that have improved our understanding of atherosclerosis and associated clinical consequences and propose model adaptations to facilitate the development of new and effective treatments.

## Introduction

Coronary artery disease (CAD), driven by the underlying pathologies of endothelial dysfunction, arterial calcification, inflammation, and atherosclerosis, is a significant risk factor for myocardial infarction and stroke. In the United States, cardiovascular disease costs >$320 billion per annum, with nearly half of the country’s adult population predicted to have cardiovascular disease by 2035 [1]. This significant health burden highlights a critical unmet need for effective pharmacotherapies to lessen the morbidity and mortality associated with CAD. Traditionally, drug therapy has targeted standard modifiable risk factors (SMuRFs) [2]. Therefore, the focus for CAD prevention has been on lipid-lowering, following the identification of a positive association between cholesterol levels and an elevated risk of myocardial infarction.

Statins, the most successful lipid-lowering drug class to date, have provided life-altering benefits to responsive patients. Yet, some patients do not tolerate statin treatment, while others remain at increased cardiovascular risk, even at the highest recommended statin dose [3]. Therefore, alternative approaches remain necessary to address this unmet clinical need, including the development of alternative lipid-lowering modalities and targeting different CAD mechanisms, such as chronic inflammation [4]. Several candidate drugs for CAD are moving through the discovery pipeline. However, the development of new medicines can be a lengthy and costly process.

In a recent global call to action for CVD drug solutions [5], several key challenges were identified in the translation of preclinical research. It was noted that the failure of many pharmacotherapies results from a lack of translation between successful preclinical studies in animal models and those conducted in humans. Unsuccessful preclinical to clinical translation can be due to undesirable drug properties, e.g. unformulated D4F designed as an apoA-I peptide mimetic had low bioavailability in humans [6], safety issues, e.g. torcetrapib (CP-529414) a cholesteryl ester transfer protein (CETP) inhibitor was terminated in Phase 3 due to disproportionate mortality in the treatment arm [7], or insufficient drug efficacy, e.g. the Phase 3 Cardiovascular Inflammation Reduction Trial (CIRT) of low dose methotrexate to reduce inflammation did not reduce cardiovascular events in patients with stable atherosclerosis [8]. Insufficient efficacy may be, at least in part, addressed by a greater understanding of the contributing mechanisms in atherosclerosis, a factor highlighted by the CIRT investigators in the context of inflammation [8]. Moreover, approaches to stratify CAD patients based on specific pathogenic mechanisms, as opposed to a common end phenotype, may enhance the translation of novel therapies [5]. Thus, bridging the preclinical to clinical gap relies on a clear understanding of the underlying biology and the implementation of preclinical models of CAD that reliably recapitulate features of the human disease.

The global call to action [5] identified potential solutions, including the identification of causal novel targets identified through the interrogation of robust animal models that mimic the human condition. The human species uniquely demonstrates a prevalence of developing CAD and experiencing a major cardiovascular event in their lifetime. Contributing factors include genetic mutations and lifestyle choices. Indeed, without intervention, whether that be environmental (diet) or genetic manipulation (gene knockout), animals are generally resistant to developing CAD, with only one documented case of a chimpanzee with CAD [9]. As such, a single *in vitro* or *in vivo* model is unlikely to accurately capture the pathophysiology or the genetic and environmental factors that contribute to the development of CAD. In this perspective, we discuss issues of translatability through the lens of reliable animal models for CAD (Table 1). Herewith, it is our intention to illuminate best practices in preclinical CAD drug discovery. Specifically, we evaluate the ideal criteria required to best recapitulate human CAD in a preclinical model. Additionally, we draw on past experiences to facilitate the future design of drug classes to improve outcomes for patients with CAD. We also clarify the hurdles currently facing CAD pharmacotherapy development and identify possible directions for future CAD drug design.

**Table 1:** Animal models of atherosclerosis: advantages and limitations

Atherosclerosis model	Advantages	Limitations	Reference
**Rabbit models**			
HCD	*Rabbits have CETP, thus a lipid profile that more closely resembles human	*Lesions resemble fatty streaks *Longer-term HFD leads to hepatotoxicity and inflammation *Larger size of animal *Higher costs of housing than mice *4–8 months to develop lesions	[31,195]
HCD plus mechanical injury	*Rapid development of lesions (2–4 weeks)	*Balloon injury of the iliac artery requires high level surgical skills *Procedure ∼45 min per animal	[32]
CRISPR/Cas9 generated LDLR deficiency	*Severe hypercholesterolemia and atherosclerosis on regular chow *Increased plasma TC, LDL-C, TG, reduced HDL *Aortic and coronary artery atherosclerosis detected	*CRISPR/Cas9 technology, zygote micro-injection and embryo transfer for generation	[196]
**Mouse**			
C57Bl/6 plus HFD	*Lesions develop in ∼15–20 weeks in aortic sinus and proximal aorta	*Primitive fatty streak lesions *Restricted distribution of lesions mostly within sinus aorta	[197]
*ApoE^−/−^* on regular chow	*Elevated cholesterol levels of 400–600 mg/dl vs wild-type mice (75–110 mg/dl) *Spontaneous lesions that are more advanced *12 weeks: fatty streaks *32 weeks: aortic plaque.	*Plasma cholesterol carried by VLDL and chylomicrons particles, differs from humans (LDL) *No plaque rupture or thrombosis	[198,199]
*ApoE^−/−^* plus HFD	*Elevated cholesterol of 1000 mg/dl *Extensive accelerated atherosclerosis in aortic sinus, aortic branches and carotid artery *Large plaques by 14 weeks	*Plasma cholesterol carried by VLDL and chylomicrons particles, differs from humans (LDL) *No plaque rupture or thrombosis	[37,200]
*Ldlr^−/−^* on regular chow or HFD	*Elevated cholesterol of 200–300 mg/dl on chow diet, and ∼1000 mg/dl on an atherogenic diet *Moderate atherosclerosis after 12 weeks of HFD *Early lesion development in proximal aorta, and distal aorta when more advanced *Better resembles human disease as cholesterol transported by LDL *Preferred model to study reverse cholesterol transport	*Do not develop spontaneous lesions on regular diet *No plaque rupture or thrombosis	[39,201]
*ApoE^−/−^* x human apoB-100 Tg or *Ldlr^−/−^* x human apoB-100 Tg	*On ApoE^−/−^ background, increased TG content *Similar serum cholesterol levels to ApoE^−/−^ mice but develop larger plaques *On Ldlr^−/−^ background: complex lesions, increased TC, TG, apo(a), reduced HDL on chow diet	*Additional crosses to these mice become difficult as homozygosity is required at 3 loci	[44,202]
*ApoE^−/−^* x *Ldlr^−/−^* dKO	*Develop coronary artery disease and myocardial infarction *Rapid plaque formation *Fibroatheromas and fibroproliferative calcified and complicated lesions develop over time (16–80 weeks) *Vasa vasorum can be studied in this model – develops extensive arterial and venous networks *Adventitial inflammation present		[203]
ApoE3-Leiden	*Reduced clearance of triglyceride-rich lipoproteins such as chylomicron- and VLDL-remnants	*No plaque rupture or thrombosis	[51]
ApoE3-Leiden.CETP	*Cholesterol profile that more closely resembles human profile *‘Humanized’ plaque profile-5 stages observed	*No plaque rupture or thrombosis	[55]
PCSK9-AAV	*Single injection and generated quickly compared with conventional crossbreeding *No confounding effects from the lack of ApoE or LDLR *Can be used to study calcification and plaque regression	*Possible antiviral host immune response	[204,205]
**Co-morbid mouse**			
*ApoE^−/−^* Ob/ob (CAD and diabetes)	*Leptin hormone deficient model – gives rise to spontaneous Type II diabetes on chow diet *Clear advantage – does not require chemical induction of diabetes	*Mice become obese *Immunometabolic dysfunction *Increased infection susceptibility	[46,56]
*ApoE^−/−^* Db/db (CAD and diabetes)	*Point mutation in gene for leptin receptor *Model of obesity, Type II diabetes and dyslipidemia *Mice more diabetic than ob/ob	*Mice become obese *Immunometabolic dysfunction *Increased susceptibility to infections	[206,207]
*Ldlr^−/−^* ob/ob (CAD and diabetes)	*Obese, hyperglycemic, hypercholesterolemic and spontaneous lesions development on chow diet	* Mice become obese *Immunometabolic dysfunction *Increased susceptibility to infections	[208]
*ApoE^−/−^Gpx1^−/−^* dKO (atherosclerosis and oxidative stress)	*Atheromas throughout aortic tree (arch to iliac bifurcation) on HFD for 24 weeks, most in abdominal aorta *Can be made diabetic with streptozotocin (STZ): lesions in sinus and aortic tree *Diabetic inflammatory changes observed after 5–10 weeks, preceding atherosclerosis *Well defined plaque with necrotic core, infiltrating inflammatory cells	*Model takes 20–24 weeks to develop *No plaque rupture observed	[57,58,209]
Tanden stenosis *ApoE^−/−^* (plaque rupture)	*Plaque rupture including thin fibrous cap, large necrotic core, intraplaque hemorrhage and plaque inflammation	*Requires microsurgery skills to suture carotid artery in two places	[59,61,62]
*ApoE^−/−^* +HFD + AngII (4wks)	*Model of hypertension, oxidative stress, increased inflammation and plaque rupture	*Implanted mini-osmotic pumps required to infuse AngII	[62]
*ApoE^−/−^Fbn1^C1039G+/−^*	*Model of spontaneous intra-plaque microvessels, hemorrhages, spontaneous plaque rupture, myocardial infarction and sudden death *Coronary plaques	*Sudden death on HFD *Cerebral blood flow disturbed; head tilt, disorientation, motor disturbances (66% of cases)	[63,210]
*ApoE^−/−^* and TAC	*Coronary plaques, myocardial infarction on a chow diet *moderate hypercholesterolemia *Plaque rupture	*Mice can die on exposure to physical stress (∼70%)	[64]
**Rat models**			
HFD	*Ease of blood collection, dissection of vessels *Easier to house than pigs, rabbits *Larger biological samples than from mice	*Less responsive to cholesterol than mice *Physiologically different metabolism and microbiome vs humans	[211]
*ApoE^−/−^* via TALEN technology	*Plasma cholesterol increased 7.6-fold vs WT rats after 8-week HFD *Mild aortic but severe coronary atherosclerosis *Myocardial cholesterol ester deposition	*Do not develop spontaneous atherosclerosis, unlike *ApoE^−/−^* mice *Require HFD to induce plaque	[212]
Sprague Dawley *ApoE^−/−^* via CRISPR/Cas9	*Lesions in males are more advanced than females, analogous with human pathology *Lesions develop in absence of HFD; model can be used to study lipid-independent effects *Heavy plaque burden after 40 weeks HFD *Severe atherosclerosis widely distributed including carotid arteries after 64 weeks of HFD	*Length of time to develop atherosclerosis	[68,213]
*Ldlr^−/−^* + HFD	*HFD: severe hyperlipidemia, lipid deposit throughout aortic tree	*Females: more severe lesions, opposite to humans	[70]
*Ldlr^−/−^* (ZFN technology)	*Plaques on HFD	*No plaques on normal diet *Develop obesity, may confound results	[70,71]
SD *Ldlr^−/−^* via CRISPR/Cas9	*Marked hyperlipidaemia, elevated cholesterol on normal chow with lesions of different stages *Heavy plaques after 40 weeks on HFD	*Liver steatosis noted	[68]
Diabetic Zucker Fatty (ZDF)	*Thoracic and abdominal lesions at 18 weeks of age *Most severe in males		[74]
CETP Tg (on a Dahl, salt-sensitive hypertensive stain)	*Develop lesions, on normal chow *Coronary plaque *Myocardial infarctions noted		[214]
Tg[hCETP] SHRs	*Hypertension, insulin resistance	*No increase in plasma LDL *Do not develop atherosclerotic plaque	[77]
**Zebrafish**			
HFD	*Major advantages: small size, low maintenance costs, large offspring numbers *Lipid metabolism comparable with humans *CETP system functional *Useful model for early progression analysis *HFD induces oxidized lipoproteins and hypercholesterolemia with early-stage lesions	*Plasma lipid profiles differ between zebrafish and humans *Small size limits certain measures: e.g. only small quantity of blood can be obtained from zebrafish ≥45 days old. *No advanced stage lesions are observed	[78]
*Ldlr* mutant by CRISPR/Cas9	*Regular diet: moderate hypercholesterolemia *High cholesterol feeding of larvae: lipids increased in blood vessels, exacerbated hypercholesterolemia and lesions	*Limitations on size and sampling as noted above apply *No advanced stage lesions are observed	[80]
*apoc2* mutant via TALEN	*Severe triglyceridemia *Larvae on normal diet: lipid accumulation in macrophages similar to humans	*Limitations on size and sampling as noted above apply *No advanced stage lesions observed	[81]
*lxr* mutant via TALEN	*LDL increase with HCD or HFD *Severe hypercholesterolemia and hepatic steatosis *Lipid accumulation and fatty streaks	*Limitations on size and sampling as noted above apply *No advanced stage lesions are observed	[11]
**Pigs**			
CRISPR/Cas9-mediated *ApoE^−/−^* and *Ldlr^−/−^* dKO	*Elevates serum LDL-C and TC levels	*High costs, long time for model development, complex experimental procedures *Lesions not reported	[87,88]
CRISPR/Cas9-mediated *ApoE^−/−^* using SCNT technology	*Moderate elevated serum cholesterol levels on chow diet *HFHC diet for 3 months led to hypercholesterolemia (both LDL and HDL) *Spontaneous human-like atherosclerotic lesions in aorta and coronaries after 6 months of HFHC (fibrous plaque)	*Time to develop lesions is long (6 months) *Plaque rupture and thrombosis not studied	[87]
**Non-human Primates**			
Cynomolgus monkeys fed a HFD for 9 months	*Ultrasound: increase in plaque in carotid (CCA and BIF) *Level of TC, TC/HDL, LDL correlated with amount of plaque and negatively correlated with HDL	*High costs, long time needed for model development *Variable responses by individuals	[91]

Abbreviations: AngII, angiotensin II; BIF, carotid bifurcation; CCA, common carotid artery; HCD, high cholesterol diet; HDL, high density lipoprotein; HFD, high fat diet; HFHC, high fat, high cholesterol diet; LDL-C, low density lipoprotein-cholesterol; NC, normal chow; TAC, transverse aortic constriction; TC, total cholesterol; TG, triglyceride; SCNT, single cell nuclear transfer; Tg, transgene; VLDL, very-low-density lipoprotein; n.i., no information; N/A, not applicable.

## Experimental models for early phase drug discovery: enhancing clinical translation by understanding the advantages and limitations

The translation of findings from preclinical studies to humans is challenging. Often it can take years before a beneficial outcome is established. Given the considerable overlap between human and mouse genomes, the results of mouse studies are frequently used for ‘go/no go’ decisions before conducting large-scale clinical cardiovascular trials. Therefore, there is a strong need to ensure that the preclinical data for the progression into clinical trials is robust to maximize clinical success and minimize the risk of failure. Equally important is the reverse, where evidence ‘in humans’ such as drug interactions are fed back into preclinical studies to improve target selection and validation. The importance of using human data to inform and improve preclinical design has been highlighted when considering sex as a biological variable [10]. Sex and age influence the incidence and complications of atherosclerosis, with sexual dimorphisms in the timing, burden, and inflammation of atherosclerotic plaques. Considering preclinical models, such as *ApoE^−/−^* and *Ldlr^−/−^* mice, gender can influence atherosclerosis development, with young (<6 months) female mice generally displaying larger plaques and greater overall burden, an effect decreased or reversed with age [10]. To date, preclinical studies typically do not use both sexes, and when they do, studies are often underpowered to statistically evaluate sex differences. It is now best practice for preclinical studies to use both sexes as default. Moreover, in humans, the influence of sex on cardiovascular disease can reverse with age, with younger women being protected while older women have a higher incidence of myocardial infarction when compared to age-matched men. Consideration should be given to these additional biological variables when designing preclinical studies.

Over the last century, the mechanisms underpinning the atherosclerotic processes have become clearer. This has, in part, been facilitated by using ever-evolving cell-based and pre-clinical animal models, often designed to model a specific biological process associated with the disease. Recently, Vedder et al. reviewed the number of PubMed publications on the topic of atherosclerosis over the period 1925–2020 [11]. The timeline reveals an initial reliance on the rabbit, followed by the rat, and since the early 1990s, with the development of knockout technologies (*ApoE^−/^*^−^, *Ldlr^−/−^*), the mouse has become the preferred strain. Interestingly, since early 2000, the use of Zebrafish has been on the rise with the ability to create knockouts via newer technologies like clustered regularly interspaced short palindromic repeat-(CRISPR)-associated protein-9 (CRISPR/Cas9) and transcription activator-like effector nuclease (TALEN). With the increasing number of species available and manipulation of the genome becoming easier, choosing the most reliable model is more challenging. Here we critique the various models, highlighting their advantages and limitations (Table 1).

### Cell-based assays

Current target-based and phenotypic drug discovery commence with *in vitro* pharmacological evaluation. Following target identification, early-stage development of new therapeutics such as small molecule drugs involves the discovery and lead optimization phases. Purified protein or recombinant cell lines are typically used for high-throughput screening to evaluate large chemical libraries, followed by hit-to-lead and lead optimization. Traditional drug discovery programs began with a high-throughput screen (HTS), sampling a defined section of chemical space for biological activity in well-defined cellular models, such as recombinant cell systems modified to express the human target of interest [12]. HTS compound libraries contain up to 3 million compounds [13]. The incorporation of computational virtual ligand screening libraries can extend the chemical sampling for a potential ‘hit compound’ into the tens of billions of compounds [13]. However, both approaches represent only a fraction of the possible >10^60^ molecules in chemical space [14]. The development and application of artificial intelligence models may provide the opportunity to explore chemical space and *in silico* design of new molecules. Computational approaches can also be useful to predict and remove compounds that are unlikely to translate into humans, based on synthetic complexity, activity, toxicity, pharmacokinetics, and unwanted drug–drug interactions [14].

Structure–activity and structure–function relationships generated through iterative candidate generation and experimental evaluation, provide a wealth of information. These relationships facilitate the delineation of the molecular mechanisms underpinning drug–receptor interactions, which in turn informs drug candidate optimization. Refining potential new therapeutics during lead optimization requires modification of candidate compounds to optimize pharmacological parameters, such as affinity and efficacy, and pharmacokinetic parameters, such as adsorption and metabolism [12,15]. The therapeutic benefit or adverse effect profile may benefit from approaches that provide greater subtype and spatiotemporal selectivity or are fine-tuned through refining the degree of agonist efficacy [16]. Indeed, partial agonists for Liver X receptor (LXR) β are being explored as an approach to stimulate therapeutic signaling whilst minimizing unwanted effects [17,18].

Beyond drug candidate profiling, primary or patient-derived cells provide a robust, and typically medium-throughput approach, to define the mechanism of action of candidate therapeutics. The generation of therapeutically relevant data is crucial during the lead optimization phase. Commonly used two-dimensional cell models for *in vitro* mechanism of action studies in the cardiovascular medicine space include endothelial cells, immune cells, and smooth muscle cells. Endothelial cells, such as human aortic endothelial cells (HAECs) and coronary artery endothelial cells, can respond to, and participate in, inflammatory events. For example, *in vitro* assays were instrumental in demonstrating that exposure of endothelial cells to the inflammatory marker C-reactive protein caused an increase in the expression of endothelin-1, adhesion molecules, alongside a decrease in endothelial nitric oxide synthase [19–21]. Collectively these findings provide a mechanistic understanding of the ability of C-reactive protein to promote proatherogenic processes, including inflammation and endothelial dysfunction. Macrophage polarization and lipid uptake models can be used to evaluate inflammatory transition and foam cell formation, respectively. For example, cultured primary mouse macrophages have identified potential mechanisms underpinning the uptake and dysregulation of macrophage lipid metabolism, and subsequent cholesterol-induced macrophage death, a typical feature of atherosclerotic lesions [22–24]. Many of these mechanisms continue to be explored *in vivo* [22]. Smooth muscle proliferation and phenotype assays can evaluate the cell phenotype changes from contractile to synthetic.

The development of multi-cellular 3D tissue-engineered models, derived from human-induced pluripotent stem cells, combine advances in tissue engineering and microfabrication technologies. 3D models applied to atherosclerosis research include spheroids, tissue-engineered blood vessels, and vessel-on-a-chip [25], which vary in terms of complexity and ability to model relevant disease processes. A recent study generated arteriole-scale tissue-engineered blood vessels, shown to reproduce features of early-stage atherosclerosis *in vitro* [26]. These perfused tissue-engineered blood vessels consisted of three cell layers, endothelial cells (inner layer), smooth muscle cells (middle layer) and fibroblasts (outer layer). Features of early-stage atherosclerosis observed in response to perfusion of enzyme-modified LDL, TNFα and/or monocytes, include vessel dysfunction, an increased inflammatory phenotype, including endothelial cell and monocyte activation, and foam cell formation. Such models highlight the potential for tissue engineering to enhance our understanding of human pathophysiology and rapidly progress the drug discovery pipeline by employing physiologically relevant *in vitro* models [27,28], even though complex tissue-engineered models cannot fully recapitulate human atherosclerosis. Addressing common obstacles encountered for *in vitro* atherosclerosis models (reviewed in [25,29]) will enhance the benefit to therapeutic development and clinical translation. In combination with animal models of CAD that robustly mirror human pathobiology, advances in preclinical *in vitro* models provide the opportunity to overcome many of the current challenges of preclinical-to-clinical translation.

### Rabbit models of atherosclerosis

The rabbit was the first effective animal model of atherosclerotic cardiovascular disease developed and was subsequently used to identify a causal link between cholesterol and atherosclerosis. Early rabbit studies demonstrated that a high cholesterol diet caused intimal thickening and aortic atherosclerosis [30]. Lesion types varied depending on the level of cholesterol feeding. Coronary artery lesions occurred in approximately 50% of rabbits fed diets greater or equal to 0.15% cholesterol. Lesions are rich in macrophages with evidence of fibrous formation [31]. Rabbits have since been used extensively in cardiovascular research.

There are clear advantages to using the rabbit, including similarities between the myocardium of the rabbit and that of humans. Rabbits are among the few species with CETP and have a lipoprotein profile that more closely resembles a human than that of rodents. The New Zealand White rabbit is most often used to study atherogenesis after high fat diet feeding. However, this requires 4–8 months of feeding to develop lesions. Several limitations, such as the high cost of housing rabbits, the significant time required for plaque formation, and the lack of genetic tools to manipulate the rabbit genome, have dampened enthusiasm for this model. A recent experimental improvement to the model is helping overcome the time it takes to develop plaque. To accelerate lesion development, a diet rich in fats (8.6%) and cholesterol (1%) can be combined with mechanical vessel injury, such as that caused by a balloon catheter to injure the endothelial layer of the left iliac artery. Atherosclerosis in this model is thought to be driven by inflammatory responses to injury. This method of inducing lesion formation significantly speeds up the process of atherogenesis, and lesions are visible within 2 weeks after injury [32].

### Mouse models of atherosclerosis

The most commonly used animal in CVD research is the mouse. With easy handling and housing, short gestation times, and tractability for genetic manipulation, these advantages make mice highly appropriate for studies evaluating a range of candidate compounds. The most significant disadvantage of mouse models is the relevance of the observed results to humans. Differences in the cardiovascular anatomy and hemodynamic forces between humans and mice influence the site of atherosclerotic lesion development [27]. Human atherogenic lesions are most prevalent in coronary and carotid arteries and peripheral vessels, whilst mouse lesions are primarily present in the aortic sinus and the aortic arch [33]. However, the most significant difference between humans and mice lies in the regulation of lipoprotein metabolism. This affects the susceptibility to the development of atherosclerosis and is due primarily to genetic differences in lipid and cholesterol processing. Hypercholesterolaemic humans have increased low density lipoprotein-cholesterol (LDL-C) levels. In rats and mice, cholesterol is mainly in the protective high-density lipoprotein (HDL) fraction due to the absence of CETP, accompanied by low-density lipoprotein (LDL) and very-low-density lipoprotein (VLDL) cholesterol levels. Thus, many small animal models require speciality diets high in lipid, cholesterol and saturated fat (known as a high fat diet) and mainly consisting of ∼15% saturated fat and 1.25% cholesterol with 0.5% sodium cholate to induce an experimental model of atherosclerosis [34]; for a comprehensive review of diets to cause atherosclerosis, refer to Table 2 of Getz and Reardon [35]. In wild-type mice on a C57Bl/6 background, this diet induces a primitive form of atherosclerosis, consisting primarily of fatty streaks in the aortic root and proximal aorta [36]. These fatty streaks consist mainly of macrophage-derived foam cells and do not generally evolve into more complex lesions. Indeed, mice require cholesterol levels of approximately 2000 mg to develop atherosclerotic lesions, levels that far exceed those commonly present in humans.

To circumvent many of these issues, genetically modified murine models have been produced to increase and/or accelerate atherogenic processes and better mimic human disease. The most commonly used atherosclerosis-prone models are the *ApoE^−/−^* and *Ldlr^−/−^* deficient mice, which have five times and 2–3 times higher plasma cholesterol levels than controls, respectively [37]. These mice develop complex, widespread plaque on a low-fat diet (4–6% fat and cholesterol content of <0.02% (w/w)) consisting of foam cells, necrotic cores and fibrous caps composed of smooth muscle cells [38,39]. In addition, advanced atherosclerosis can be induced by varying the diet in these mice, where sodium cholate is often omitted. Dietary modifications, including fructose addition to induce insulin resistance and alterations to lipid and cholesterol content, have allowed for more in-depth analyses of their contributions to atherosclerosis development [40]. Rapid and robust atherosclerotic lesion formation can also be induced in *ApoE^−/−^* mice through partial carotid ligation, leading to disturbed flow profiles [41]. The ease of obtaining and breeding these two genetically altered mouse strains has led to their widespread use. This, in turn, has strengthened their utility since their lesions have been well described by many and appear within a well-defined time frame [42]. Most importantly, atherosclerotic lesions are morphologically comparable to human lesions [37].

In addition to these, numerous genetically modified models have been developed for cardiovascular experimental purposes [43]. These include mice overexpressing the human apoB-100 transgene fed a high-cholesterol, cholate-containing diet. This transgene increases the TG content and contributes to increased atherosclerosis throughout the aortic tree [44]. The use of dietary sucrose in *Ldlr^−/−^ ApoB^100/100^* has highlighted the role of metabolic inflammation in inducing atherosclerosis in these mice [45]. Additional models such as the *ApoE/Ldlr* double-knockout, ApoE3-Leiden and PCSK9- adeno-associated virus (AAV) [46] mice are valuable tools in atherosclerosis research [46,47].

PCSK9-AAV mice were developed after a single injection of a recombinant adeno-associated virus containing a gain-of-function mutant form of PCSK9. Combined with a high fat diet, these mice show reduced LDLR expression, increased plasma LDL cholesterol, and advanced aortic root lesions consisting of foam cells, smooth muscle cells and fibrous tissue [46]. Extensive calcification also develops in this model [48,49], whilst replacing the diet with a regular diet for 6 weeks facilitates the study of plaque regression [50].

A recent advance on the ApoE3-Leiden mouse model was achieved by crossing these mice with mice overexpressing the CETP transgene, generating a ‘humanized’ ApoE*3-Leiden.CETP (E3L.CETP) mouse model. In this model, the CEPT transgene promotes a cholesterol profile with greater similarity to humans, whereas the ApoE*3-Leiden transgene decreases the clearance of TG-rich lipoproteins [51]. This results in a mouse model that most closely replicates human atheroma with all five stages of disease represented (type I to V). In response to drug therapies such as statins, fibrates, ezetimibe and antidiabetic medications, these mice demonstrate lipid profiles and atherosclerotic progression that resembles the human condition [52,53]. These mice have shown the efficacy of anti-PCSK9 therapies for lowering LDL-C and TGs [54], which translated to reduced atherosclerosis development in the aortic root. These mice are also an adequate model to study the effect of the metabolic syndrome after a high-fat, high-cholesterol diet for 6 months [55].

Another consideration is the genetic strain of the mouse on which to create the genetic modification. Among mouse strains, the C57Bl/6 is most often used as it is the most sensitive to the development of atheromas. Other strains of mice vary in resistance to atheroma development. A further benefit of genetically modified atherosclerosis-prone mice is the ability to generate atherosclerosis against a background of common cardiovascular comorbidities such as Type II diabetes (e.g. *ApoE^−/−^* leptin (*ob/ob*) and *ApoE^−/−^* leptin receptor (*db/db*) deficient mouse strains) [56]. In addition, the versatility of breeding strategies has allowed for the generation of additional mouse models of accelerated atherosclerosis, e.g. *ApoE/Gpx1* double knockout mice [57,58].

### Plaque rupture mouse models

One of the most significant differences between the atherosclerosis seen in humans versus mice or other animals is plaque rupture. Several mouse models have been developed recently to mirror this important feature of human CAD more closely. The development of a tandem stenosis (TS) procedure that occludes the carotid vasculature and recapitulates hemodynamic shear stress has led to a model with evidence of significant plaque rupture in *ApoE^−/−^* mice [59–61]. Plaque rupture characteristics in the lesions in animals that have undergone the TS procedure include thin fibrous caps, large necrotic cores, intraplaque hemorrhages, plaque inflammation, and neovascularization, as observed in humans. These animals also respond to pharmacological intervention with statins [59] and therefore can be used to evaluate plaque-stabilizing drugs.

Another model of plaque rupture, spontaneous atherothrombosis and aortic aneurysm is the *ApoE^−/−^* mouse fed a high fat diet for 4 weeks and infused with Angiotensin II (AngII) for a further 4 weeks. AngII elevates blood pressure, recruits and activates monocytes, and increases oxidative stress, promoting plaque rupture under hypertensive conditions [62]. Another model worth considering is the *ApoE^−/−^Fbn1^C1039G+/−^* mouse that carries a heterozygous mutation (C1039^+/−^) in the fibrillin-1 (*Fbn1*) gene, leading to fragmentation of the elastic fibres of the vessel wall. These mice consistently develop intra-plaque microvessels, spontaneous haemorrhages, plaque rupture, myocardial infarction, and sudden death [63]. In yet a further model, pressure overload can be employed to increase coronary plaque formation, progression, and myocardial infarction in *ApoE^−/−^* mice on a chow diet (moderate hypercholesterolemia) [64]. More than 90% of the mice develop atherosclerotic lesions in the coronary arteries within 8 weeks of transverse aortic constriction (TAC), with a significant portion showing stenosis of the left anterior descending artery. Most mice (∼70%) died when exposed to physical stress, with immunohistochemical evidence suggestive of coronary occlusion, plaque rupture, and/or erosion. Importantly, this study revealed that *ApoE^−/−^* mice are susceptible to coronary lesion formation. It offers a potential new model that more closely mimics the human condition of CAD to study alterations in genetic factors or drug treatments to specifically target coronary atherosclerosis.

### Rat models of atherosclerosis

There are significant advantages to using rats over mice to study cardiovascular disease. These include the ease of blood collection and dissection of blood vessels. It is also cheaper to purchase, house, feed and maintain rats than larger animals like pigs and rabbits. However, rats are generally less responsive to dietary cholesterol compared to mice, often requiring cholic acid or thiouracil-containing high cholesterol and high fat diets to cause hyperlipidemia and atherosclerosis. Other drawbacks include the physiological differences in their metabolism and microbiome, which may impact CVD drug development. A major disadvantage has been the difficulty in manipulating the genome of the rat since most of the techniques have been developed for the mouse. Thus, gene deletion studies using gene targeting technologies are limited in rats. However, recent developments have made targeted gene deletion in rats easier, with more groups attempting to create gene knockouts in rats [65].

Several recent studies report the development of *ApoE^−/−^* rats. The first study only showed very early signs of lipid deposition and atherosclerosis [66]. A second study generated an *ApoE^−/−^* rat using TALEN technology. These authors report proatherogenic dyslipidemia after a high-cholesterol diet, but the rats showed no evidence of inflammation and did not develop atherosclerotic plaque [67]. A third study used CRISPR/Cas9 technology in Sprague Dawley (SD) rats to generate *ApoE^−/−^* rats. These rats developed hyperlipidemia with decreased HDL-C, up-regulated TC and LDL-C, and plaques that resembled human atherosclerosis [68]. Endothelial inflammation was observed in these rats, as evidenced by increased atherosclerosis-related adhesive and inflammatory gene expression. Lesions developed on a normal diet, while the feeding of a Western diet significantly increased plaque development after 40 weeks, with severe widespread atherosclerosis throughout the arterial tree, including the carotids, after more prolonged feeding (64 weeks) [68]. However, a further study using *ApoE^−/−^* rats fed a high fat diet for 51 weeks only detected evidence of early atherosclerosis as assessed by increased intima/media thickness ratio and macrophage influx into *ApoE^−/−^* aortic intima [69].

LDLR deficient rats have also been generated and shown to develop aspects of atherosclerosis after a high fat diet. For example, using N-ethyl-N-nitrosourea to induce a missense mutation in exon 4, LDLR mutant rats developed hypercholesterolemia, with significant lipid deposition extending from the aortic arch to the abdominal aorta as well as in the aortic valves [70]. Interestingly, female rats had more severe lesions than males, which is opposite to that seen in humans. Using Zinc-finger nuclease (ZFN) technology, Wang et al. generated an *Ldlr^−/−^* rat with a 19 bp deletion in exon 7. These rats displayed elevated TC, TG, LDL-c and VLDL-c in their plasma, but no arterial plaques were detected on a normal diet [71]. Others have shown arterial plaques in *Ldlr^−/−^* rats using different diets or techniques. For example, a ZFN-generated rat *Ldlr^−/−^* model fed a high fat diet developed obesity and atherosclerotic plaques [72], whilst Zhao et al., using CRISPR/Cas9 technology, generated *Ldlr^−/−^* rats that displayed hyperlipidemia, inflammation, atherosclerotic lesions, and liver steatosis, particularly when on a high fat diet for 40 weeks [68]. Subtle differences were noted between the two models, mainly around HDL levels and the level of obesity achieved in the ZFN-generated *Ldlr^−/−^* rats which were not observed in CRISPR/Cas9-generated rats [65]. CRISPR/Cas9-generated *Ldlr^−/−^* rats have been crossed with CRISPR/Cas9-generated *ApoE^−/−^* rats, with male double knockout rats displaying a higher atherosclerotic plaque burden than female rats, the profile seen in human disease. Therefore, this model may be effective for studying the effect of drugs on atherosclerosis in a model more aligned with the human condition.

Various modifications of Zucker fatty (ZF) rats have been used to study obesity, diabetes, and atherosclerosis. ZF rats, which contain a missense mutation (fatty, fa) in the leptin receptor gene (*Lepr*), are a model of obesity in the absence of diabetes [73]; whereas the subsequently derived Zucker diabetic fatty (ZDF) rat is a model of obesity in the presence of diabetes [74]. ZDF rats are obese, display dyslipidemia, and show phenotypes consistent with Type 2 diabetes using oral glucose tolerance testing. Analysis of aortic en face lipid staining with Oil-Red-O to assess macrovascular disease showed atherosclerosis in the thoracic and abdominal aorta at 18 weeks of age, with the most severe atherosclerosis seen in male *ApoE^−/−^* rats. Chemical induction of diabetes via the pancreatic β-islet cell toxin streptozotocin (STZ) in Sprague-Dawley rats fed a high fat diet is another way of generating diabetic and obese mice for studying atherosclerosis [75]. Using this rat model, Li *et al*. showed the effectiveness of liraglutide, an analog of human glucagon-like peptide-1 (GLP-1), in reducing plaque burden in diabetic obese rats.

Like mice, rats are deficient in CETP, which contributes to their atherosclerosis resistance. To circumvent this, human CETP transgenic rats have been developed. In a Dahl salt-sensitive hypertensive rat model moderately overexpressing hCETP, coronary plaque was detected along with hypercholesterolemia and hypertriglyceridemia, which was worse in males compared with females [76]. More recently, a human CETP-transgenic (Tg[hCETP]) rat strain on the spontaneously hypertensive rat (SHR) background was generated. These rats displayed spontaneous hypertension and insulin resistance but failed to show increased plasma LDL cholesterol levels. Moreover, a high-fat, high-cholesterol diet did not appear to promote atherosclerosis progression in Tg[hCETP] SHRs [77].

### Zebrafish models of atherosclerosis

Another model that has drawn attention for its use in large-scale screening of anti-atherogenic drugs is the zebrafish, due to its small body size, low rearing costs and large offspring numbers [78]. While the zebrafish vascular system is different from mammals, there are commonalities across the species in vascular development and anatomy. Lipid metabolism in zebrafish is similar to that in humans, and they are proving useful for studies of lipid accumulation and lipoprotein oxidation. In particular, the fact that zebrafish have retained the evolutionarily central CETP gene has enabled the development of dyslipidemia models without difficulty [79]. Furthermore, the larvae of zebrafish are being used in drug discovery to study the effects of compounds on the development of a whole organism in a cost-effective and time-saving manner. Therefore, zebrafish have become a popular alternative to rodents and rabbits for atherosclerosis research.

Several zebrafish models have been developed, recently reviewed by Tang et al. [78]. These include a high-cholesterol diet (HCD) induced model and three genetically manipulated zebrafish models containing mutations in *Ldlr*, *apoc2, or lxr*. The HCD model shows a hypercholesterolemic phenotype with vascular lipid accumulation (early-stage lesions) and early lipoprotein oxidation [79]. *Ldlr* mutant zebrafish, created by CRISPR/Cas9 technology, show increased lipid accumulation in blood vessels, hypercholesterolemia and type II atherosclerotic lesions [80]. The LPL activator, *apoc2*, is a key enzyme involved in the processing and removing chylomicron particles from the blood. Zebrafish containing an *apoc2* mutation, created with TALEN technology, show increased chylomicrons and severe hypertriglyceridemia [81]. LXRs regulate cholesterol catabolism and fatty acid and glucose homeostasis. In zebrafish, unlike humans, only one isoform exists with similarities to LXRα. TALEN-mediated *lxr* deletion increases lipid accumulation, with lipid deposits showing similarities to fatty streaks in humans [82].

### Pig models of atherosclerosis

Large animal models such as pigs are suitable models to study atherosclerosis due to similarities in their lipid metabolism and vascular lesion morphology with humans [83]. Pigs closely mirror the lipid profiles of humans, with cholesterol being carried by LDL. Being a larger animal, features such as heart size, vasculature and anatomy of the aorta are similar to humans. Pigs develop spontaneous atherosclerotic lesions in the coronary arteries on regular chow. While the atherosclerotic process is slow, it can be enhanced by HFD and intravascular mechanical interventions to damage the vessel wall (e.g. balloons or stents) [84]. A recent study describes the technique of endovascular administration of calcium chloride to the carotid arteries of pigs to model arterial calcification. Via catheter administration, a short exposure (9 mins) resulted in extensive circumferential parietal calcification, as confirmed by imaging modalities (intravascular ultrasound and optical coherence tomography) and histological assessments [85]. Despite clear advantages, pigs are not commonly used due to limitations around size, housing, and often prohibitive costs.

Recent advances in creating genetically modified pigs are solving the low propensity to develop atherosclerosis. PSCK9 transgenic pigs have been created by transposon integration. CRISPR/Cas9 has been used to generate pigs lacking either LDLR or ApoE [84,86]. Lack of ApoE resulted in human-like atheromas in the aorta and coronaries with evidence of fibrous plaque after 6 months on a high-fat high cholesterol diet [87]. A recent study used CRISPR/Cas9 to target both LDLR and ApoE simultaneously. Minipigs showed significantly elevated levels of LDL-C, total cholesterol and apoB with no off-target effects [88]. The extent of atherosclerosis was not reported.

### Non-human primate (NHP) models of atherosclerosis

As our closest relatives from an evolutionary perspective, the common chimpanzee might be thought of as the ideal model to study the development of atherosclerosis with >99% protein sequence homology with humans [89]. Despite having levels of LDL significantly higher than that observed in humans (∼50%), analyses of coronary arteries from chimpanzees show a surprising resistance to the development of coronary plaque. Evidence suggests a heightened T-cell immune response by humans compared with chimpanzees as a possible cause of this differential response [90]. High-fat feeding of Cynomolgus monkeys led to dyslipidaemia which correlated with the level of plaque detected in the carotid arteries upon ultrasound analysis [91]. Dyslipidemic rhesus or cynomolgus monkeys have been used to study the effectiveness of mABs to *ANGPTL3 and* PCSK9 to lower LCL-C as discussed previously [92–94]. Other mAB treatments tested in monkeys include evinacumab, which caused a significant decrease in TG levels [95,96]. Newer technologies have also been tested including ASOs e.g., Volanesorsen to suppress apoC3 biosynthesis [97] and LNA ASOs targeting PCSK9 mRNA.

#### The ideal animal model

From the very small zebrafish to large non-human primates, each model listed above brings its unique qualities to the atherosclerosis landscape. The choice of a perfect model can be overwhelming. Most importantly, the choice of an ideal model should be dictated by the question being posed. For example, treatments for advanced atherosclerosis and plaque rupture can only be addressed using models with proven pathology of significant necrotic core, rupture, and thrombosis. Many of the listed models (Table 1) fail to recapitulate this basic tenet, which is now accepted as a critical driver of major adverse CVD outcomes. Furthermore, the ideal model should develop atheromas that resemble the human anatomy and align closely with the human pathophysiology underpinning atherogenesis. From a practical perspective, the animal model should be easy to purchase and/or breed, affordable to house and maintain, and easy to handle. Finally, regardless of the model that is ultimately used, rigorous experimental design practices such as relevant control groups, randomization and blinding, *a priori* sample size calculation to ensure sufficient statistical power, and appropriate statistical analyses are essential to maximize the chance of translatability of treatments into the clinic.

## Current lipid-lowering treatments for atherosclerosis: success stories, limitations, and future therapies

**Figure 1:**
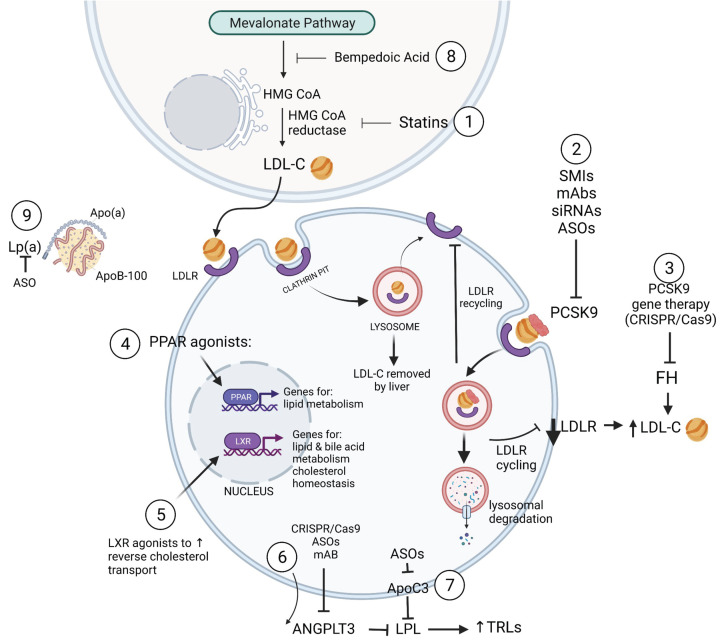
Multiple therapeutic targets exist for the treatment of atherosclerosis (1) Statins reduce LDL-C production by inhibiting HMG-CoA reductase; (2) PCSK9 inhibitors disrupt the recycling of LDLR; (3) PCSK9 gene therapy can be used to treat patients with familial hypercholesterolemia (FH); (4) PPAR agonists promote expression of lipid metabolism genes; (5) LXR agonists promote expression of lipid and bile acid metabolism genes and increase reverse cholesterol transport; (6) ANGPTL3 inhibitors counteract LPL inhibition and reduce TG-rich lipoprotein (TRL) levels; (7) ApoC3 inhibition increases LPL activation and decreases TG levels; (8) Bempedoic acid catalyses the production of acetyl-CoA, and decreases HMG-CoA formation; (9) Lp(a) inhibitors reduce LDL-C levels.

The role of lipids in CAD is well-known, and drug therapy aimed at lipid-lowering is widely considered the most effective approach for lowering the risk of mortality (Figure 1) [98]. A clear target of cholesterol-lowering has been the cholesterol biosynthesis pathway, with inhibition of the rate-limiting step enzyme, HMG-CoA reductase, the focus of the statin drugs [99]. The history of statin discovery, pre-clinical testing and translation into clinical practice is an exemplar of the numerous steps along the translational path. The role that preclinical, rigorous animal testing played in this process has been highlighted in a systematic review where 120 studies were assessed for the ability to lower total cholesterol (TC) [100]. The analysis covered 18 studies in mice, 61 in rats and 41 in rabbits, across multiple different genetic backgrounds for each species. Most studies were performed on male subjects; 64% of studies used a standard diet whereas 36% used a high-cholesterol diet. The results showed that statins lowered TC in all the species considered. However, differences in effect size were observed with rabbits showing ∼30% reduction, mice ∼20% and rats ∼10%. Reductions in TC by statins in clinical trials in humans seem to parallel that seen in rabbits (∼30%). This highlights the influence of the model on the outcome, and the need to choose carefully to optimize outcomes. It also emphasized that rabbits fed a high cholesterol diet (HCD) were the model of choice, showing a strong positive effect of statins in lowering TC, LDL-C and triglycerides (TGs), but a negative effect in raising HDL-C. It revealed an interesting conundrum; statins were more active in animals fed HC diet, inconsistent with the current understanding of the drug’s mechanism of action in inhibiting endogenous cholesterol synthesis. Furthermore, data from *in vitro* and animal studies additionally suggested that statins not only reduce LDL-C and TGs but also have other positive off-target (pleiotropic) effects. These include reductions in endothelial, oxidative and inflammatory stress, decreasing markers of platelet activation, increasing high-density lipoprotein cholesterol, improving cardiac, renal and endothelial function, and, ultimately, improving atherosclerosis [101]. The successful transition of statins from preclinical testing in animal models through to effective therapy can therefore be attributed to a clear mechanistic understanding of its *modus operandi* in inhibiting HMG-CoA reductase, conferring lipid-lowering benefits across several preclinical species, and its positive pleiotropic effects on key atherogenic pathways.

In the 1990s, the Scandinavian Simvastatin Survival Study (4S) was the first to show that cholesterol was lowered with a statin. In this case, the semi-synthetic agent simvastatin conferred a significant reduction in LDL-C values, mortality and major coronary events [102,103]. The success of this secondary prevention trial stimulated multiple primary prevention trials of statins, including the West of Scotland Coronary Prevention Study (WOSCOPS) [104] and the Collaborative Atorvastatin Diabetes Study (CARDS) trial [105]. Statins have now been tested in large-scale clinical trials that followed subjects for 5 years [103,104]. Such studies consistently demonstrate that statin treatment can effectively reduce plasma LDL-C levels by 35–55% and the frequency of myocardial infarction by 25–30% [99]. Newer statins, including atorvastatin and rosuvastatin, have increased potency for lowering LDL-C and may achieve the desired LDL-C reduction more safely [106]. Statins remain an important component of atherosclerotic cardiovascular disease prevention and remain among the top-selling class of drugs worldwide [107,108].

Despite the significant success of statin therapy, some patients are statin-intolerant and develop myalgia and myopathy, particularly at high statin doses [109,110], and many statin-treated patients retain a residual risk of a major cardiovascular event [111,112]. Indeed, modifying cardiovascular risk factors beyond traditional lipid-lowering, e.g. inflammation, may be required to address the elevated cardiovascular risk observed for many statin-treated patients [5]. Furthermore, some patients with familial hypercholesterolemia (FH) or established CAD cannot achieve optimal lipid-lowering, despite high-intensity statin treatment [3]. These limitations highlight the need for alternative well-tolerated LDL-C lowering approaches that, when used as a monotherapy or in combination with a statin, decrease the risk of CAD through mediating a significant and durable lowering of LDL-C levels.

### PCSK9 inhibition: a translational drug discovery case study

**Figure 2:**
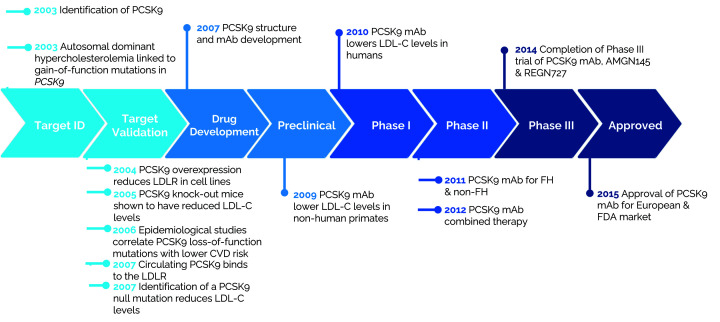
Timeline of the translation of PCSK9 inhibitors from discovery to Federal Drug Administration (FDA) approval

Proprotein convertase subtilisin/kexin type 9 (PCSK9) inhibitors were rapidly translated from discovery science into the clinic. This process required less than 5 years for target validation and a subsequent 7 years for development and federal drug administration (FDA) approval (Figure 2) [113]. PCSK9 is a serine protease that degrades the LDL receptor (LDLR). A subset of individuals with FH were shown to have PCSK9 gain-of-function mutations [114]. Conversely, PCSK9 loss-of-function mutations are associated with low cholesterol levels and a significant reduction in the incidence of CAD [115]. These genetic insights enabled the identification of a new biological target for hypercholesterolemia and coronary artery disease (CAD), which led to the development of PCSK9 inhibitors.

### PCSK9 mechanism of action

PCSK9 is primarily expressed and secreted by the liver, with lower expression in other tissues, including the intestine, kidneys, and central nervous system [116]. Diurnal variations and the fasting state influence the circulating levels of PCSK9, which are higher in women than men [98]. PCSK9 contains an LDLR binding site and decreases LDLR-mediated clearance of circulating LDL. In the absence of PCSK9, LDL particles bound to the LDLR are internalized as an LDL-LDLR complex via clathrin-coated pits into the endocytic recycling compartment. The low pH environment promotes dissociation of the LDL-LDLR complex. The LDLR then recycles back to the cell surface, while LDLs are targeted for lysosomal degradation [116]. However, internalization of LDL-LDLR complexes bound, with high affinity, to PCSK9 results in the entire ligand–receptor complex being directed towards lysosomal degradation, which prevents the LDLR from recycling back to the cell surface [116]. Degradation of LDL-LDLR-PCSK9 complexes decreases LDL-C removal from circulation.

Preclinical studies demonstrated that PCSK9 modulates LDL-C levels through loss- and gain-of-function studies in genetically manipulated mice. Studies in PCSK9-deficient, atherosclerotic mouse models have shown significantly reduced accumulation of cholesteryl esters in the aorta. These mice had a 2.8-fold increase in hepatic LDLR expression and a marked reduction in total plasma cholesterol [117]. In contrast, overexpression of PCSK9 induced excess cholesterol accumulation, leading to atherosclerosis [118]. PCSK9-mediated increases in the progression of atherosclerosis are LDLR-dependent, as the effect of PCSK9 knockdown or overexpression on cholesteryl ester accumulation and plaque size is abolished in LDLR-deficient mice [119]. Clearly, these preclinical studies have identified a significant role for PCSK9 inhibition in LDL-C lowering, but whether this translates into improvements in plaque stabilization remains to be definitively proven in preclinical models. Encouraging results using near-infrared spectroscopy in patients on PCSK9 inhibitors show that lipid-rich vulnerable coronary plaques are stabilized by reducing the lipid core burden index [120].

Several drug development strategies are under evaluation for pharmacological inhibition of PCSK9 based on these results, including gene silencing with small interfering RNAs (siRNAs) or antisense oligonucleotides (ASOs), small molecule inhibitors and well as monoclonal antibodies (mAbs) [117].

#### Clinically approved PCSK9 inhibitors

##### Monoclonal antibodies (mAbs)

Throughout the development of mAb targeting PCSK9, mAb efficacy for PCSK9 inhibition was assessed in small and large animal models. A specific PCSK9 mAb, alirocumab, administered as a single intravenous infusion in mice, caused a significant and dose-dependent reduction in circulating non-high-density lipoprotein cholesterol (HDL-C) levels [121]. Moreover, in humanized mice and non-human primates, a second high-affinity mAb recognising the LDLR-EGF(A) binding domain of PCSK9 caused a dose-dependent reduction in LDL-C [122]. In rhesus monkeys, PCSK9 mAbs significantly lower LDL-C levels [123]. When combined with simvastatin, a PCSK9 mAb successfully lowered LDL-C in monkeys with metabolic syndrome and had similar effects in primates fed a high fat diet [93,124]. A modified mAb engineered to escape degradation through increased pH sensitivity was as potent in reducing total cholesterol levels as the original mAb in mice and monkeys, decreasing cholesterol levels 75 days post-injection [94].

Two large-scale clinical trials were undertaken to evaluate two PCSK9 mAbs for effects on cardiovascular outcomes. The PCSK9 inhibitor, Evolocumab caused a substantial reduction of LDL-C levels and a relative reduction in cardiovascular events, even beyond that seen with statin therapy in The Further Cardiovascular Outcomes Research with PCSK9 inhibition in Subjects with Elevated Risk (FOURIER) study [125]. A second trial, the ODYSSEY OUTCOME trial, evaluated the effects of Alirocumab and observed significant reductions in major adverse cardiovascular events (MACE) and almost a 90% reduction in LDL-C levels despite these patients already receiving high-intensity statin therapy [126]. Participants in both trials were considered high-risk with highly elevated LDL-C levels. The trial demonstrated the effectiveness of PCSK9 inhibition even when combined with a high-intensity statin therapy [127].

Trials with FDA-approved anti-PCSK9 mAbs have established that PCSK9 inhibition effectively decreases LDL-C levels. However, these therapies are associated with complex manufacturing requirements and a prohibitively high-cost [128,129], >100-times the cost of generic statins in the United States [130]. Assuming similar efficacy across different LDL-C levels, an economic evaluation of trial data found PCSK9 inhibitors were only cost-effective in 1 of 24 patient subgroups. Collectively these factors have resulted in persistently low uptake of these treatments, even in high-income countries [130].

##### Small interfering RNA (siRNA)

While inhibitory monoclonal antibodies that target PCSK9 are now in use in clinical practice, there are ongoing efforts to develop different, more economical approaches for inhibiting PCSK9. Inclisiran, approved for use in numerous counties, is a first-in-class siRNA therapeutic for LDL-C reduction, acting to silence PCSK9 translation in the liver [131]. Preclinical animal models demonstrated 50% and 70% inhibition of PCSK9 in both rodent and non-human primates, respectively [132]. Successful Phase I studies with Inclisiran did not identify side effects of concern. Inclisiran lowers LDL-C by ∼50% or greater for 6 months, allowing twice-yearly administration to improve long-term lipid-lowering in high-risk patients. Indeed, a 50% reduction in LDL-C levels was observed following 3–6 monthly Inclisiran injections in Phase III trials of atherosclerotic CVD patients, including those with increased LCL-C levels despite maximal statin therapy [133]. Ongoing Phase III studies are evaluating the long-term efficacy and safety of Inclisiran. These extensive 5–6 year studies are double-blind, randomized placebo-controlled trials assessing the effects of Inclisiran on MACE in patients with established CVD (ClinicalTrials.gov: NCT03705234, NCT05030428).

#### PCSK9 inhibition approaches currently under development

##### Antisense oligonucleotide (ASO) inhibition of PSCK9

ASOs, typically 15 to 30 nucleotide length single-stranded molecules, can alter gene expression by two mechanisms, they can occupy the target mRNA and prevent it from being translated, or they can induce degradation of the target mRNA. Second-generation ASOs targeting PCSK9 mRNA evaluated in preclinical studies demonstrated an increase in LDLR expression and an associated reduction in LDL-C and apoB-100 levels in mice fed a high fat diet [134]. However, development was terminated due to relatively low binding affinity. Focus shifted to shorter ASOs using locked nucleic acid (LNA) ASO technology with a more stable conformation and increased selectivity for PSCK9 [127]. LNA ASOs targeting PCSK9 mRNA levels conferred an increase in the hepatic expression of LDLR in mice and a reduction in plasma LDL-C and apoB levels in monkeys [135,136]. Further development of ASO technology has seen the emergence of an orally available ASO targeting PCSK9, currently undergoing Phase I testing. Taken up by the liver, this oral ASO showed 5–7% bioavailability compared with subcutaneous injections in rats and dogs, with a reduction in LDL-C observed in healthy monkeys.

##### PCSK9 vaccines

PCSK9 vaccines, such as AT04A and AT06A, are under development as an alternative, more cost-effective approach to the mAb-based therapies [137]. Immunization with PCSK9 peptide-based vaccines can initiate a strong anti-PCSK9 immune response, significantly decreasing PCSK9 and LDL-C levels in murine models. Moreover, this approach has decreased systemic and vascular inflammation alongside atherosclerotic development in APOE*3Leiden.CETP mice [138]. Two peptide vaccine candidates targeting PCSK9, recently evaluated in a Phase I study in healthy patients, were safe and initiated an immunogenic response. One candidate mediated a significant reduction in LDL-C levels [139].

##### PCSK9 gene therapy

Individuals with homozygous PCSK9 loss-of-function mutations and a consequent lack of circulating PCSK9 protein are healthy and have a deficit of LDL-C [117]. Knocking down the *Pcsk9* gene using technologies such as CRISPR/Cas9 has effectively disrupted the PCSK9 gene in CRISPR-*Pcsk9* mice [140]. The efficiency of this approach is evidenced by decreased circulating PCSK9, increased hepatic expression of LDLR, and a 35–40% reduction in total cholesterol in CRISPR-*Pcsk9* mice. Similarly, genome editing *Pcsk9* in mice using an adeno-associated virus vector significantly reduced circulating PCSK9 and total cholesterol levels one-week post-injection [141]. PCSK9 gene editing has also been applied to larger animal models. The first study in rhesus macaques observed both on-target and off-target effects, which persisted (albeit less) after reengineering the adeno-associated virus. On-target effects mediated a dose-dependent reduction in PCSK9 and LDL-C [142]. Additional studies performed in cynomolgus macaques used lipid nanoparticles for *in vivo* delivery of CRISPR adenine base editors targeting PCSK9 [143,144]. This approach appeared to address the previous limitation of unwanted off-target effects. The longer study reported a 60% reduction in LDL-C at 8 months, whereas the shorter study had lower efficacy of base editing and only a 14% reduction in LDL-C at 29 days in macaques. To date, PCSK9 gene therapy has yielded promising results in animal models. However, long-term safety data in non-human primates are required before potential application in humans.

##### Small molecule PCSK9 inhibitors

Small molecule inhibitors of PSCK9 are being explored as a mechanism to develop cost-effective and orally available therapeutics. Small molecule development has employed structural biology, molecular modeling, and high throughput screening approaches. Several synthetic and naturally occurring small molecules that inhibit PCSK9 have been identified [114]. Target mechanisms include direct interaction with the PSCK9–LDLR interface or interference with the synthesis, maturation, or secretion of PCSK9. While small molecule inhibitors are an attractive therapeutic approach, a current barrier when targeting the PCSK9–LDLR interface is the deficit of clear druggable binding pockets. The PCSK9–LDLR protein–protein interaction has a relatively flat surface on both the PSK9 and LDLR binding domains and a secondary structure resistant to small molecule interactions [145]. Future studies could explore the development of small molecule compounds targeting a conformationally-linked but spatially distinct allosteric binding pocket to inhibit PCSK9–LDLR interactions [16].

### Other lipid-lowering therapies

#### Bempedoic acid

Alternative approaches for inhibiting cholesterol biosynthesis have been explored, including targeting the step before HMG-CoA reductase in the liver. Bempedoic acid (8-Hydroxy-2,2,14,14-tetramethylpentadecanedioic acid, also known as ETC-1002) is an oral inhibitor of hepatic ATP citrate lyase, involved in hepatic cholesterol synthesis. Bempedoic acid acts like a statin by upregulating hepatic expression of the LDLR and decreasing plasma LDL-C levels.

Bempedoic acid confers beneficial effects in mouse models of diet-induced metabolic dysregulation. In *Ldlr^−/−^* mice fed a high-fat, high-cholesterol diet, bempedoic acid-mediated a dose-dependent decrease in TGs, cholesterol, hyperinsulinemia, fatty liver, and obesity. Moreover, bempedoic acid prevented inflammatory gene expression and atherosclerotic lesion development [146]. Similar anti-inflammatory effects of bempedoic acid were observed in a subsequent study using diet-induced obesity in mice. In this study, bempedoic acid had a beneficial effect on systemic inflammation with reduced expression of the macrophage-specific marker F4/80 [147].

Effects of bempedoic acid on cholesterol synthesis were observed in clinical trials, demonstrating a favourable decrease in LDL-C, an effect more pronounced when administered as a co-therapy with ezetimibe in statin-intolerant patients [148]. Interestingly, the anti-inflammatory properties of bempedoic acid observed in animal models were not evident in clinical trials. Bempedoic acid has moved through clinical trials across different patient populations, and as of February 2020, the FDA granted approval for this agent. Bempedoic acid is indicated in patients with hypercholesterolemia or established atherosclerotic cardiovascular disease as an adjunct therapy to diet and maximally tolerated statin treatment.

#### ANGPTL3 inhibitors

Angiopoietin-like 3 (ANGPTL3) is a secretory protein produced in the liver [149]. It regulates the metabolism of triglyceride-rich lipoproteins and plasma lipid levels by inhibiting TG hydrolysis by lipoprotein lipase (LPL) and phospholipid hydrolysis by endothelial lipase. Loss-of-function ANGPTL3 variants, identified in the DiscovEHR human genetics collaboration, were associated with lower TG and LDL-C levels and a 41% lower risk of CAD [95]. ANGPTL3 regulates the production and clearance of apolipoprotein (apo) B-containing lipoproteins.

Preclinical models of atherosclerosis have consistently shown beneficial effects with decreased ANGPTL3 expression. In ApoE deficient mice, reduced expression of ANGPTL3 elevated catabolism and clearance of TG-rich lipoproteins, which conferred protection against hyperlipidemia and atherosclerosis [150]. ASO silencing of ANGPTL3 reduced plasma TGs, HDL-C, and LDL-C levels in dyslipidemic mice (wild-type C57BL/6 mice, *Ldlr^−/−^* mice, *Apoc3^−/−^ Ldlr^−/−^* mice, heterozygous *Apoc3^+/−^ Ldlr^−/−^* mice, diet-induced obese mice, mice overexpressing human APOC3) [151,152]. These effects were most profound in LDLR knockout mice, where treatment with the ANGPTL3 ASO slowed the progression of atherosclerotic lesions [151]. Adenoviral CRISPR/Cas9 silencing of ANGPTL3 in wild-type and hyperlipidemic *Ldlr^−/−^* mice *in vivo* caused a significant reduction in plasma ANGPTL3, TGs and cholesterol. In this study, an estimated 35% gene inactivation resulted in a >50% decrease in plasma TGs and LDL-C in *Ldlr^−/−^* mice [92]. The LDL-C reduction observed following ANGPTL3 inhibition or decreased expression appears to be independent of the LDLR, as these effects remain in *Ldlr^−/^*^−^ mice [153].

Pharmacological inhibition of ANGPTL3 confers similar therapeutic effects, highlighting the potential to prevent atherosclerosis progression. Treatment of dyslipidemic mice and monkeys with evinacumab, a high affinity, fully humanized monoclonal antibody against ANGPTL3, significantly reduced TG levels [95,96]. In mice, evinacumab reduced TG levels by 84% and atherosclerotic plaque size by 39% [95]. Evinacumab has been assessed in clinical trials for hypercholesterolemia, hypertriglyceridemia and homozygous familial hypercholesterolemia. A Phase II study of evinacumab in patients with refractory hypercholesterolemia despite maximally tolerated lipid-modifying therapy demonstrated a >50% reduction in LDL-C in patients treated with the maximal dose [154]. Positive trial results were also obtained in a Phase III study of evinacumab in patients with homozygous familial hypercholesterolemia taking maximally tolerated lipid-modifying therapy. In these patients, evinacumab reduced LDL-C by 49% [155]. Evinacumab is approved as an adjunct therapy in patients with homozygous familial hypercholesterolemia. Like antibodies against PCSK9, this agent is expensive, and its use will likely be hampered by cost.

#### PPARδ agonists

Peroxisome proliferator-activated receptors (PPARs) are a family of nuclear receptor proteins that, when activated, serve as transcriptional regulators of target genes, including those involved in lipid metabolism, oxidative stress, and inflammation [156]. Thiazolidinediones are potent PPARγ agonists used to treat diabetes mellitus, while fibrates are selective PPARα agonists used to treat dyslipidemia [156]. PPARδ is ubiquitously expressed and suggested to regulate lipid homeostasis. The potent and selective PPARδ agonist, GW-501516, has been evaluated *in vitro* and *in vivo*. These studies have provided some insight into the complex role of PPARδ, particularly in terms of lipid metabolism, suggesting an important regulatory role for PPARδ with regard to fatty acid oxidation [157]. In obese rhesus monkeys, GW-501516 conferred significant beneficial effects, specifically an increase in HDL-C and a decrease in LDL-C [158]. A small-scale clinical trial observed a GW-501516 mediated reduction in total cholesterol and TG levels. However, concerns exist regarding the tumor-promoting effects of PPARδ activators, including GW-501516 [159]. A recently identified rationally designed potent and selective PPARδ small molecule selectively modulates PPARδ activity [160,161]. As such, these pharmacological tools provide the opportunity to explore the role of PPARδ in lipid metabolism and oncogenesis, providing a better understanding of targeted PPARδ therapeutic potential.

#### LXR agonists

Liver X receptors (LXRs) are ligand-activated transcription factors that significantly regulate lipid and bile acid metabolism and cholesterol homeostasis. LXR agonists increase reverse cholesterol transport and, therefore, may inhibit atherosclerosis progression [162]. However, hepatic steatosis and increased VLDL secretion, leading to hypertriglyceridemia, has been observed upon oral administration of LXR agonists in mice [156]. In addition, LXR stimulation elevated LDL-C, particularly in species that express cholesteryl ester transfer protein (CETP), such as non-human primates [163]. Of the two subtypes of LXR agonists, LXRα is primarily expressed in the liver and increases hepatic lipogenesis. In contrast, LXRβ is ubiquitously expressed, and activation can reverse atherosclerosis and cholesterol accumulation in peripheral tissues in ApoE knockout mice that are also LXRα deficient [164].

LXR-623 is a highly selective and orally bioavailable synthetic agonist for LXRβ. A preclinical study of non-human primates with normal lipid levels saw this LXRβ modulator significantly decrease total cholesterol and LDL-C in a time- and dose-dependent manner [163]. In contrast, however, the first human clinical trial using ascending doses of LXR-623 saw the two highest doses cause central nervous system-related adverse events. As such, LXR-623 development was discontinued [162]. CS-8080 and BMS-852927 were two other synthetic agonists terminated due to safety concerns or undisclosed reasons [165]. Partial LXRβ selective agonists, BMS-779788 and IMB-808, have improved lipid profiles in non-human primates and *in vitro* screening, respectively [17,18]. As such, partial agonists of LXRβ may provide an alternative avenue for developing LXR agonists as potential atherosclerosis therapeutics with an acceptable side effect profile.

#### ApoC3

Apolipoprotein C3 (apoC3) can promote atherosclerosis through modified lipid metabolism. ApoC3 inhibits LPL, which increases TG levels. ApoC3 levels are positively associated with CAD risk and are higher in patients with Type 2 diabetes [166]. Humans with apoC3 loss-of-function mutations have lower TG levels and raised LPL activity, associated with a reduced CV risk [167]. Overexpression of human apoC3 in mice results in severe hypertriglyceridemia and atherogenesis [168,169]. In a recent preclinical study, apoC3 antisense oligonucleotides reduced atherogenesis and lowered TGs. Whilst specific apoC3 small molecule inhibitors are not in development, a high throughput screen of almost 1 million molecules identified a series of retinoic acid receptor agonists that prevented the inhibition of LPL by apoC3 [170].

The specific ASO inhibitor Volanesorsen, which suppresses apoC3 biosynthesis has been developed and evaluated in rodents (mice models: C57BL/6, *Ldlr^−/−^*, Ob/Ob, *apoC3^−/−^*, CETP transgenic *Ldlr*^−/−^; rat models: Sprague-Dawley, Zucker diabetic fatty) and hypertriglyceridemic rhesus monkeys [97]. The influence of apoC3 inhibition was consistent across animal models, causing a significant reduction in the known cardiovascular risk factors, plasma apoC3 and TG levels [97].

#### Lp(a)-lowering agents

Lipoprotein(a) (Lp(a)) is composed of an LDL with an apolipoprotein(a) (apo(a)) covalently bound to apolipoprotein B-100 (apoB). Lp(a) is a strong independent, causal, genetic risk factor for CAD, involved in atherosclerotic and calcific aortic valve disease and a promising biomarker for CAD risk assessment [171,172]. New therapeutics designed to lower Lp(a) levels are currently under development. ISIS-301012 (mipomersen) is a selective ASO inhibitor of apoB-100, reducing Lp(a) assembly [171]. Preclinical characterization of the mouse apoB100-specific ASO, ISIS-147764, was performed in mouse models of hyperlipidemia (C57BL/6 high fat diet, *Ldlr*-deficient, *ApoE*-deficient). In these studies, ISIS-147764 produced a significant, dose-dependent reduction in apoB100 and LDL-C levels without causing hepatic steatosis [173]. An *in vivo* pharmacokinetic/pharmacodynamic study of a series of 2′-O-(2-methoxyethyl) modified ASOs identified a consistent exposure-response relationship across species (mice, monkeys and humans), supporting the ability of animal models to predict human dosing for hepatic-targeted ASOs [174]. Following successful clinical trials, ISIS-301012 gained approval for the treatment of individuals with homozygous familial hypercholesterolemia.

## Inflammation-targeted therapies

Inflammation is a causal factor for human atherosclerosis. Chronic inflammation is underpinned by persistent activation of innate and adaptive immune responses. Treatments targeting chronic inflammation provide a critical alternative or adjunct approach to established lipid-lowering therapies. Several inflammatory mechanisms are gaining traction as new therapeutic targets for CAD.

### Initial studies *in vitro* and in the rabbit

The link between high fat diets, inflammation, and atherosclerosis was first established in rabbits. Atherosclerotic plaques were demonstrated in high fat diet fed New Zealand rabbits, Watanabe heritable hyperlipidemic rabbits with a genetic LDLR deficiency, and transgenic rabbits expressing human genes implicated in lipid metabolism and atherosclerosis [30]. Plaques from these rabbit models contained cells expressing the proinflammatory cytokine interleukin (IL)-1β. The mechanism by which high cholesterol within atherosclerotic plaques causes IL-1β processing and release was established *in vitro* [175]. Intraplaque cholesterol crystals were shown to act as damage-associated molecular patterns (DAMPs) to directly activate the NLRP3 (nucleotide oligomerization domain-, leucine-rich repeat-, pyrin domain-containing protein 3) inflammasome.

### Mouse studies of inflammation

Mouse studies have been pivotal in demonstrating the involvement of various members of the inflammasome pathway in atherogenesis. The development of atherosclerotic lesions was observed to decrease in LDL-R deficient mice fed a high fat diet when transplanted with bone marrow deficient in NLRP3, ASC, IL-1β, Il-18 or IL-1α, highlighting the importance of the inflammatory pathway in the development of atherosclerosis [175–177]. However, *ApoE^−/−^* mouse models of atherosclerosis have generated some controversy around the role of the NLRP3 inflammasome. Initial studies in *NLRP3/ApoE*, *ASC/ApoE* and *caspase-1/ApoE* double knockout mouse models failed to reveal significant effects on atherosclerosis, plaque macrophage numbers or stability. These studies suggested atherosclerosis develops independently of NLRP3 inflammasome activation [178]. However, atheroprotection was observed in *caspase-1/ApoE* double knockout mice fed a high fat diet [179]. Experimental design, the microbiota environment and different cholesterol diet formulations may have contributed to the observed differences in atherosclerosis progression.

Small molecule inhibitors have firmly established the significance of the NLRP3 inflammasome in the atherogenic process. The selective small-molecule inhibitor, MCC950, administered for 4 weeks to high fat diet *ApoE*^−/−^ mice, significantly reduced the atherosclerotic plaque burden [180]. This reduction in plaque was also demonstrated under diabetic conditions, where MCC950 administered intraperitoneally as an interventional approach improved vascular function and led to a more stable plaque phenotype containing a decreased complement of inflammatory cells [181]. Several inhibitors of the NLRP3 pathway are currently in drug development. In addition, a significant reduction in atherosclerotic lesion size has been observed upon administration of the IL-1 receptor antagonist, anakinra [182–184].

### Human trials

#### Anti-IL-1β antibodies (CANTOS)

The highly anticipated large-scale multicentred Canakinumab Anti-inflammatory Thrombosis Outcome (CANTOS) Phase III trial involved 10,061 patients with previous myocardial infarction and elevated C-reactive protein. This clinical trial tested the efficacy of a monoclonal antibody, canakinumab, that neutralizes IL-1β. It showed a significant reduction in inflammation without affecting lipid levels and a significantly lower rate of recurrent cardiovascular events (15%, *P*=0.021) at 150 mg of canakinumab, highlighting a causal link between inflammation and cardiovascular events. Importantly, this was the first human trial to prove the inflammation hypothesis of atherosclerosis. The path leading to this landmark study included preclinical development *in vitro* and preclinical animal studies. *In vitro* studies established the specificity of canakinumab for human Il-1β whilst failing to engage with human IL-1α or rodent Il-1β. Mouse studies showed the neutralization of human IL-1β function in models of arthritic joint inflammation [185]. Phase I/II double-blind, randomized dose-escalating trials were undertaken in rheumatoid arthritis (RA) patients, confirming a mean half-life of 21.5 days [185]. Numerous other clinical trials were undertaken prior to CANTOS for indications relating to auto-inflammatory syndromes such as gout, RA, cryopyrin-associated periodic syndromes, systemic-onset juvenile idiopathic arthritis, chronic obstructive pulmonary disease (COPD), Type 2 diabetes and wet age-related macular degeneration [186]. This highlights that the road to clinical development can be lengthy and often not a direct path for CAD indication. To date, canakinumab has not been approved for secondary prevention of cardiovascular events [187]. Limitations of canakinumab use for cardiovascular disease include the association of anti-inflammatory treatment with a higher incidence of fatal and non-fatal infections than placebo [188] and economic analysis demonstrated that canakinumab is not cost-effective for secondary prevention of cardiovascular events at the current market price [189].

#### Colchicine

A traditional anti-inflammatory medicine, colchicine, has been identified as a potential therapy to reduce cardiovascular events [190]. Colchicine’s modes of action include inhibition of tubulin polymerization and microtubule generation, with suggested effects on the NLRP3 inflammasome, cellular adhesion molecules and inflammatory chemokines. Animal models exploring the NLRP3-mediated anti-inflammatory effects of colchicine have revealed inhibition of caspase-1, IL-1β, P2X7 receptor and the Mediterranean fever gene [190]. Colchicine treatment also improves endothelial cell function and decreases inflammation independent of lipid levels in hyperlipidemic rats [191]. Unlike canakinumab, colchicine is relatively inexpensive and is orally administered.

Colchicine has been studied in a range of cardiovascular conditions [190]. The Low-Dose Colchicine (LoDoCo) trial of 532 patients with stable coronary disease revealed a significant decrease in cardiovascular events upon treatment with 0.5 mg once daily dose of colchicine, relative to placebo treatment [192]. The subsequent, larger COLCOT trial of 4745 patients, which was randomized, double-blind, placebo-controlled and investigator-initiated, identified a significant decrease in the risk of death from cardiovascular causes such as myocardial infarction or stroke or urgent hospitalization for angina leading to coronary revascularization in patients receiving 0.5 mg of colchicine per day, compared with those receiving placebo [193]. In this study, colchicine reduced the primary endpoint from 7.1% in the Placebo group to 5.5% in the treated group.

### Co-morbidities and polypharmacy

Cardiovascular disease prevalence increases along with multiple comorbidities such as obesity, diabetes, and chronic kidney disease. With the ever-increasing ageing population, multiple cardiovascular drugs are often prescribed to improve mortality and morbidity [194]. However, polypharmacy increases the risk of inappropriate drug interactions and adverse outcomes. Preclinical animal models rarely take these issues into account for several reasons: (1) most models cannot recapitulate the multiple comorbidities that are commonplace in humans; (2) drug metabolism and drug interactions can often not be replicated in an animal model; (3) it is expensive to evaluate the effect of a potential drug in multiple models.

## Future perspectives for atherosclerotic drug discovery

Despite initial advances in the treatment of atherosclerosis via statin therapies (i.e. targeting modifiable risk factors), it is now evident that current pharmacotherapies that focus on decreasing LDL-C levels are not sufficient for a substantial proportion of patients that progress to recurrent acute events despite optimal LDL-lowering treatment. A broader understanding of the mechanisms underpinning CAD pathobiology, such as recognition of the role of chronic inflammation, holds great promise for identifying new molecular pathways that can be therapeutically targeted [4,28]. Most importantly, preclinical models of CAD that better mimic the human condition with respect to plaque structure and rupture, will facilitate enhanced efficacy and reduced toxicity of new pharmacotherapies. The development of newer rodent models that mimic plaque rupture is one way to address these limitations. Collaborative efforts in building efficient discovery platforms e.g. integrated ‘omics’ approaches, and the use of consistent cell-based and animal models of CAD, with well-defined common endpoint measurements, will enhance the success of pharmacotherapies transitioning from preclinical studies to human clinical trials. Additional streamlining to improve the time and cost of preclinical to clinical development would incentivize industry investment and bolster the drug discovery pipeline, progressing candidate compounds that are more likely effectively translate into humans.

## Data Availability

This manuscript is a review article and therefore, data sharing is not applicable.

## Open Access

Open access for this article was enabled by the participation of Monash University in an all-inclusive *Read & Publish* agreement with Portland Press and the Biochemical Society under a transformative agreement with CAUL.

## Abbreviations

AAV: adeno-associated virus
AngII: Angiotensin II
ANGPTL3: Angiopoietin-like 3
apo: apolipoprotein
apoC3: Apolipoprotein C3
ASO: antisense oligonucleotide
CAD: coronary artery disease
CETP: cholesteryl ester transfer protein
COPD: chronic obstructive pulmonary disease
CRISPR: clustered regularly interspaced short palindromic repeat
CRISPR/Cas9: CRISPR-associated protein 9
CVD: cardiovascular disease
DAMP: damage-associated molecular pattern
FDA: federal drug administration
FH: familial hypercholesterolemia
GLP-1: glucagon-like peptide-1
HAEC: human aortic endothelial cell
HCD: high-cholesterol diet
HDL: high-density lipoprotein
HTS: high-throughput screen
IL: interleukin
LDL: low-density lipoprotein
LDL-C: low density lipoprotein-cholesterol
LDLR: low density lipoprotein receptor
LNA: locked nucleic acid
LPL: lipoprotein lipase
LXR: Liver X receptor
mAb: monoclonal antibody
MACE: major adverse cardiovascular events
NHP: Non-human primate
NLRP3: nucleotide oligomerization domain-, leucine-rich repeat-, pyrin domain-containing protein 3
PCSK9: Proprotein convertase subtilisin/kexin type 9
PPAR: peroxisome proliferator-activated receptor
RA: rheumatoid arthritis
SD: Sprague Dawley
SHR: spontaneously hypertensive rat
siRNA: small interfering RNA
SMuRF: standard modifiable risk factor
STZ: streptozotocin
TAC: transverse aortic constriction
TALEN: transcription activator-like effector nuclease
TC: total cholesterol
TG: triglyceride
TS: tandem stenosis
VLDL: very-low-density lipoprotein
ZDF: Zucker diabetic fatty
ZF: Zucker fatty
ZFN: Zinc-finger nuclease
